# Alternative empirical Bayes models for adjusting for batch effects in genomic studies

**DOI:** 10.1186/s12859-018-2263-6

**Published:** 2018-07-13

**Authors:** Yuqing Zhang, David F. Jenkins, Solaiappan Manimaran, W. Evan Johnson

**Affiliations:** 1 Division of Computational Biomedicine, Boston University School of Medicine, 72 East Concord Street, Boston, 02118 MA USA; 2 Graduate Program in Bioinformatics, Boston University, 24 Cummington Mall, Boston, 02215 MA USA; 3 Department of Biostatistics, Boston University School of Public Health, 715 Albany Street, Boston, 02118 MA USA

**Keywords:** Batch effects, Empirical Bayes models, Data integration, Biomarker development

## Abstract

**Background:**

Combining genomic data sets from multiple studies is advantageous to increase statistical power in studies where logistical considerations restrict sample size or require the sequential generation of data. However, significant technical heterogeneity is commonly observed across multiple batches of data that are generated from different processing or reagent batches, experimenters, protocols, or profiling platforms. These so-called batch effects often confound true biological relationships in the data, reducing the power benefits of combining multiple batches, and may even lead to spurious results in some combined studies. Therefore there is significant need for effective methods and software tools that account for batch effects in high-throughput genomic studies.

**Results:**

Here we contribute multiple methods and software tools for improved combination and analysis of data from multiple batches. In particular, we provide batch effect solutions for cases where the severity of the batch effects is not extreme, and for cases where one high-quality batch can serve as a reference, such as the training set in a biomarker study. We illustrate our approaches and software in both simulated and real data scenarios.

**Conclusions:**

We demonstrate the value of these new contributions compared to currently established approaches in the specified batch correction situations.

**Electronic supplementary material:**

The online version of this article (10.1186/s12859-018-2263-6) contains supplementary material, which is available to authorized users.

## Background

In the past two decades, owing to the advent of novel high-throughput techniques, tens of thousands of genome profiling experiments have been performed [1, 2]. These massive data sets can be used for many purposes, including understanding basic biological function, classifying molecular subtypes of disease, characterizing disease etiology, or predicting disease prognosis and severity. Initially, the majority of these studies were completed using microarray platforms, but sequencing platforms are now common for many applications. Across these platforms, thousands of variations on technology and annotation have been used [3]. Scientists who seek to integrate data across platforms may experience difficulty because platforms introduce distinct technological biases and produce data with different shapes and scales. For example, gene expression microarrays typically measure transcription levels on a continuous (log-) intensity scale, whereas RNA-sequencing measures the same biological phenomena with overdispersed and zero-inflated count data. Furthermore, even with data from the same platform, large amounts of technical and biological heterogeneity is commonly observed between separate batches or experiments. Due to the high cost of these experiments or the difficulty in collecting appropriate samples, datasets are often processed in small batches, at different times, and in different facilities. This proves to be a difficult challenge to researchers wanting to combine studies to increase statistical power in their analyses.

One illustrative example of cross-platform (and within-platform) heterogeneity can be found in The Cancer Genome Atlas (TCGA) [4]. Profiling data types collected by TCGA include RNA expression, microRNA expression, protein expression, DNA methylation, copy number variation, and somatic mutations. Within each profiling type, multiple platforms are often used. For example, RNA expression has been measured with RNA-sequencing (using multiple different protocols) and several different microarray platforms including Agilent G4502A, Affymetrix HG-U133A, and Affymetrix Human Exon 1.0 ST, among others. Many of the tumor samples are profiled by only a subset of the possible data types and platforms, and in almost all cases the samples within each platform were generated in multiple experimental batches. This presents problems to researchers wanting to do comprehensive and integrative analyses, as they often limit their analyses to a single data type or platform. Furthermore, other existing data resources (e.g. ENCODE, LINCS, Epigenome Roadmap) utilize different platforms and protocols, and researchers often want to combine their own experimental data with data from these public repositories. Therefore, these necessitate robust and sophisticated standardization and batch correction methods in order to appropriately integrate data within and across these consortiums.

Many prior studies have clearly established the need for batch effect correction [5, 6]. To address these difficulties, existing tools have been developed for batch correction. Some of the first batch effect methods relied on singular value decomposition (SVD) [7], machine learning classification approaches (DWD) [8], or a block linear model (XPN) [9]. The SVD approach relies on the identification of batch effects by the unsupervised matrix decomposition, which will commonly result in the removal of biological signal of interest. The DWD and XPN methods provide supervised approaches for combining data, but are mostly used to combine two batches at a time, and do not account for treatment effects. These methods need to be applied multiple times in an ad hoc manner for studies with three or more batches, and are not flexible enough to handle multiple experimental conditions, or studies with unbalanced experimental designs. More recent and flexible methods rely on robust empirical Bayes regression (ComBat) [10], the efficient use of control probes or samples (RUV) [11], or more sophisticated unsupervised data decomposition (SVA) [12] to remove heterogeneity from multiple studies while preserving the biological signal of interest in the data, even when the experimental design across the studies are not balanced.

However, despite these useful existing approaches for data cleaning and combination, there are still significant gaps that need to be addressed for certain data integration scenarios. For example, the ComBat approach removes batch effects impacting both the means and variances of each gene across the batches. However, in some cases, the data might require a less (or more) extreme batch adjustment. Below, we present higher order moment-based metrics and visualizations for evaluating the extent to which batch effects impact the data. Then, at least in the case of less severe batch effects, we propose a simplified empirical Bayes approach for batch adjustment.

Another limitation of the current ComBat model is that it adjusts the data for each gene to match an overall, or common cross-batch mean, estimated using samples from all batches. While this approach is advantageous for cases with small sample size or where the batches are of similar caliber and size, this is not the best solution when one batch is of superior quality or can be considered a natural ’reference’. In addition, the current ComBat approach suffers from sample ’set bias’ [13], meaning that if samples or batches are added to or removed from the set of samples on hand, the batch adjustment must be reapplied, and the adjusted values will be different–even for the samples that remained in the dataset in all scenarios. In some cases, the impact of this set bias can be significant. For example, consider a biomarker study, where a genomic signature is derived in one study batch (training set) and then later applied or validated in future samples/batches (test sets) which were not collected at the time of biomarker generation. Once the test sets are obtained and combined with the training data using ComBat, the post-ComBat training data may change and the biomarker may need to be regenerated. Many statistical tests that are commonly used for biomarker derivation, such as t-test and F-test, involve calculating data variance. As ComBat adjustment reduces or expands the variance for each gene, it will result in a different test statistics, followed by an increased or reduced P value. This may cause certain genes to be included or excluded from the biomarker list, resulting in a different biomarker from before. If ComBat is applied on multiple training/test combinations separately (i.e. say at different times), then the derived biomarker may be different between different dataset combinations. Therefore, the value of establishing the training set as a ’reference batch’ to which all future batches will be standardized would have a significant impact. This would allow the training data and biomarker to be fixed a priori but still enable the application of the biomarker on an unlimited set of future validation or clinical cohorts.

Although these alternative models for batch only represent alternative formulations of the original ComBat modeling approach, their implementation will have significant downstream impacts on certain batch combination scenarios. Below, we detail these modifications and demonstrate their utility and increased efficacy on real data examples.

## Methods

We present several approaches for improved diagnostics and batch effect adjustment for certain batch adjustment situations. We focus on developing models based on the ComBat empirical Bayes batch adjustment approach, although similar methods and models can be applied to other existing approaches. One set of diagnostic procedures attempts to characterize the distributional (mean, variance, skewness, kurtosis, etc) differences across batches. We present a solution for the cases where adjusting only the mean of the batch effect is sufficient for harmonizing the data across batches. In addition, we present an approach that allows the user to select a reference batch, or a batch that is left static after batch adjustment, and to which all the other batches are adjusted. This approach makes sense in situations where one batch or dataset is of better quality or less variable. In addition, this approach will be particularly helpful for biomarker studies, where one dataset is used for training a fixed biomarker, then the fixed biomarker is applied on multiple different batches or datasets, even at different times. This approach avoids the negative impacts of test set bias in the generation of the biomarker signatures. Below we describe the methodological developments for these cases.

### ComBat batch adjustment

ComBat [10] is a flexible and straightforward approach to remove technical artifacts due to processing facility and data batch. ComBat has been established as one of the most common approaches for combining genomic data across experiments, labs, and platforms [14], and has been shown to be useful for data from a broad range of types and biological systems [15, 16]. The ComBat batch adjustment approach assumes that batch effects represent non-biological but systematic shifts in the mean or variability of genomic features for all samples within a processing batch. ComBat assumes the genomic data (*Y*_*ijg*_) for gene *g*, batch *i*, and sample *j* (within batch *i*) follows the model:

1 $$\begin{array}{@{}rcl@{}} Y_{ijg} = \alpha_{g} + X_{ij}\beta_{g} + \gamma_{ig} + \delta_{ig}\varepsilon_{ijg} \end{array} $$

where *α*_*g*_ is the overall gene expression. *X*_*ij*_ is a known design matrix for sample conditions, and *β*_*g*_ is the vector of regression coefficients corresponding to *X*_*ij*_. *γ*_*ig*_ and *δ*_*ig*_ represent the additive and multiplicative batch effects of batch *i* for gene *g*, which affect the mean and variance of gene expressions within batch *i*, respectively. The error terms, *ε*_*ijg*_, are assumed to follow a normal distribution with expected value of zero and variance $\sigma _{g}^{2}$. ComBat assumes either parametric or nonparametric hierarchical Bayesian priors in the batch effect parameters (*γ*_*ig*_ and *δ*_*ig*_) and uses an empirical Bayes procedure to estimate these parameters [10]. This procedure pools information across genes in each batch to shrink the batch effect parameter estimates toward the overall mean of the batch effect empirical estimates. These are used to adjust the data for batch effects. This approach provides a robust and often more accurate adjustment for the batch effect on each gene.

### Moment-based diagnostics for batch effects

The ComBat model described above robustly estimates both the mean and the variance of each batch using empirical Bayes shrinkage, then adjusts the data according to these estimates. However, in some cases, adjusting only the mean of the batches may be sufficient for further analysis. In other scenarios (see examples in “” below), adjustment of the mean and variance is not sufficient, and thus the adjustment of higher order moments is needed. Here, we present multiple diagnostics for interrogating the shape of the distribution of batches to determine how batch effect should be adjusted.

As with ComBat, we assume that in the presence of batch effect, the mean and variance (as well as higher order moments) of gene expression demonstrate systematic differences across batches on a standardized scale [10]. Thus, we standardize the data as we have done previously, namely by estimating the model (1) above, obtaining the estimates for the parameters and calculating the standardized data, *Z*_*ijg*_, as follows:

2 $$\begin{array}{@{}rcl@{}} Z_{ijg} = \frac{Y_{ijg} - \hat{\alpha}_{g} - X_{ij}\hat{\beta}_{g}}{\hat{\sigma}_{g}} \end{array} $$

After standardization, we assume the standardized data, *Z*_*ijg*_, originate from a distribution with mean *γ*_*ig*_, variance $\delta _{ig}^{2}$, skewness *η*_*ig*_, and kurtosis *ϕ*_*ig*_. In addition, consistent with the ComBat assumptions, we assume that each of these moments originates from a common distribution (henceforth denoted the *hyper-distribution*), namely that the *γ*_*ig*_ are drawn from a distribution with mean *γ*_*i*_ that is common across all genes. Similar assumptions of exchangeability across genes are made about the variance, skewness, and kurtosis.

We apply two tests of significance to individually test for significant differences in these moments across batches. In both of the tests, we estimate and conduct the test on the hyper-moments (i.e. moments of the hyper-distribution) across batches. The first test estimates the hyper-moments within each sample, whereas the other test estimates the hyper-moments within each gene. The first, sample-based test is more robust for small sample size, whereas the second, gene-based test is more robust and sensitive in larger sample size. Finally, for quantile-normalized data [17], the sample-wise test will fail because quantile normalization will naturally force all moments to be the same across samples. So for quantile normalized data, the gene-wise test will be needed.

#### Sample-level moments

The first test is a sample-level test that estimates the hyper-moments by summarizing the moments of gene expression within each sample, and then conducts a standard or *robust* F-test (described below) to compare the moment estimates across batches. For example, for the mean, the sample-wise test first estimates the mean gene expression of each sample, namely

3 $$\begin{array}{@{}rcl@{}} \bar{\gamma}_{ij} = \frac{1}{n_{g}}\sum_{g}Z_{ijg} \end{array} $$

where *n*_*g*_ is the total number of genes. We then conduct an F-test on the $\bar {\gamma }_{ij}$ values between batches. Similarly, the variance, skewness, and kurtosis of the *Z*_*ijg*_ are estimated across genes within each sample (using standard estimation approaches for these moments) and then tested for significant differences across batches in the same way as the mean. Overall this is not specifically testing the moments of the assumed ComBat model hyper-distribution, but rather the marginal distribution of the data and hyper-distributions of the data.

#### Gene-level moments

The second test is a gene-level test that estimates the hyper-moments within each gene, using samples in each batch separately, and then conducts a robust F-test comparing the moment estimates across batches. For example, for the mean, the gene-wise test first estimates the mean of each gene across samples within a batch, namely

4 $$\begin{array}{@{}rcl@{}} \bar{\gamma}_{ig} = \frac{1}{n_{i}}\sum_{j}Z_{ijg} \end{array} $$

where *n*_*i*_ is the total number of samples in batch *i*. We then conduct a test of significance on the $\bar {\gamma }_{ig}$ values between batches. Similarly, the variance, skewness, and kurtosis of the *Z*_*ijg*_ are estimated across samples within each batch for each gene (using standard estimation approaches for these moments) and then tested for significant differences across batches in the same way as the mean. Unlike the sample-wise test, this test more specifically follows the ComBat hierarchical model assumption, by first estimating the parameters drawn from the hyper-distribution.

#### Robust F-test

In hypothesis testing, a large enough sample size may cause the *P*-value problem [18]: small effects with no practical importance can be detected significant, as the *P*-value quickly drops to zero under a very large sample size. The *P*-value problem influences tests that are sensitive to the sample size, including the F-test used in this study. To address this problem and better interpret the results of the two tests above, we applied a robust F-test. The robust F-test is modified from standard F test, by adding a variance inflation factor in the F statistics, which accounts for the influence of the sample size in *P*-values. Details of the robust F-test are documented in Additional file 1.

The robust F-test is especially useful for the gene-level test, as the total degrees of freedom in this test is in fact the amount of moment estimates, which equals to the number of genes times the number of batches. This value can easily become very large in genomic studies. We used both the robust and the non-robust versions of F statistics in the sample- and gene-level tests, and evaluated and compared their performances in diagnosing the degree of batch effect in our example data.

### Mean-only adjustment for batch effects

The current ComBat model adjusts for effects in both the mean and the variance across batches. However, for some datasets, after testing for the moments of the batch effect, it may be determined that differences are only present in the mean across batches. Other datasets may be expected to have variance differences across batches for non-technical reasons, such as in a study combining a in vitro perturbation experiment (low variance) with patient samples (high variance). For cases in which that batch differences are only present in the mean, we have modified the current ComBat model to only adjust the mean batch effect. Specifically, we modify the ComBat model (1) as follows:

5 $$\begin{array}{@{}rcl@{}} Y_{ijg} = \alpha_{g} + X_{ij}\beta_{g} + \gamma_{ig} + \varepsilon_{ijg} \end{array} $$

and then use the same approach for standardization and shrinkage as described previously with the exception of not estimating and adjusting for variance differences across batches.

### Reference batch adjustment

Many batch adjustment approaches, including ComBat, are dependent on the datasets in hand for their batch adjustments. In other words, if additional samples or batches of data are added, the batch adjustments and adjusted data would be different. We present a reference-based batch adjustment approach that uses one batch as the baseline for the batch adjustment. The reference batch is not changed and the other batches are adjusted to the mean and variance of the reference. Thus, as long as the reference batch does not change, the adjustments and adjusted data would be the same, regardless of the batches of data that are included in the dataset. This also allows batches of data to be adjusted at different times without impacting the results. This approach will be advantageous to data generating consortiums where data arrive sequentially in small batches. It will also be important for applications in personalized medicine where biomarkers need to be established and validated prior to the collection of patient data. For our reference-based version of ComBat, we will assume a model slightly different than the model (1) presented above, namely:

6 $$\begin{array}{@{}rcl@{}} Y_{ijg} = \alpha_{rg} + X_{ij}\beta_{rg} + \gamma_{rig} + \delta_{rig}\varepsilon_{ijg} \end{array} $$

where *X*_*ij*_ and *β*_*rg*_ are the design matrix and regression coefficients as described before, but *α*_*rg*_ is the average gene expression in the chosen reference batch (*r*). Furthermore, *γ*_*rig*_ and *δ*_*rig*_ represent the additive and multiplicative batch differences between the reference batch and batch *i* for gene *g*. The error terms, *ε*_*ijg*_, are assumed to follow a normal distribution with expected value of zero and a reference batch variance $\sigma _{rg}^{2}$. The empirical Bayes estimates for *γ*_*rig*_ and *δ*_*rig*_ will be obtained as in the current ComBat approach.

### Software implementation

The models presented here have been integrated into the ComBat function available in the ’sva’ Bioconductor package (version 3.26.0) [12, 19]. More specifically, ComBat now includes optional parameters ’mean.only’, which if TRUE will only adjust the mean batch effect and not the variance, and ’ref.batch’, which allows the user to specify the batch name or number to be used as the reference batch. Our moment-based diagnostic tests for the mean, variance, skewness, and kurtosis are now available in our ’BatchQC’ Bioconductor package [20]. BatchQC is an R software package designed to automate many important evaluation tasks needed to properly combine data from multiple batches or studies. BatchQC conducts comprehensive exploratory analyses and constructs interactive graphics for genomic datasets to discover the sources of technical variation that are present across multiple sets of samples. BatchQC currently provides both the supervised diagnostics for known sources of technical variation (data generating batch, reagent date, RNA-quality, etc) as well as an unsupervised evaluation of batch effects to detect unmeasured non-biological variability or ’surrogate variables’ [12].

### Dataset descriptions

#### Pathway simulation

We generated simulated data to represent a case where we (1) derive a gene expression signature of a biological pathway or drug perturbation, and (2) profile the signature into another batch of data to predict pathway activity (or drug efficacy). The study consists of two experimental batches which are designed as follows: batch 1 is given by a 200 (gene) by 6 (sample) matrix of expression data, where the columns contain three replicate samples before pathway activation and three after activation (i.e. overexpressing key pathway driving genes). Among the 200 genes, the first 100 represent ’signature genes’ that are differentially expressed (before vs. after) based on a ’before’ Gaussian distribution: *N*(0,0.1), and an ’after’ distribution: *N*(1,0.1). The rest of the genes are drawn from a *N*(0,0.1) distribution in all 6 samples, representing genes that do not respond to the pathway perturbation. Batch 2 consists of a 200 (gene; same genes as batch 1) by 600 (sample) matrix, and represents a large and highly variable patient data set. The 600 patients are divided equally into 6 subgroups with different levels of pathway activation between groups; signature genes are drawn from a *N*(*μ*,10) distribution, where *μ*=0.5,0.7,0.9,1.1,1.3, and 1.5 for the six subgroups. The control genes are drawn from a *N*(0.5,10) distribution. We set up these simulation studies based on the design of real signature profiling studies [21], and selected parameters to capture the statistical properties of realistic gene expression distributions (Additional file 2). Simulation code for this dataset is available at https://github.com/zhangyuqing/meanonly_reference_combat.

#### Bladder cancer

We used a previously published bladder cancer microarray dataset, which aims to measure gene expression in superficial transitional cell carcinoma (sTCC) in the presence and absence of carcinoma in situ (CIS) [22]. This dataset contains 57 observations generated at 5 different processing times. It was previously established that the processing time is strongly confounded with CIS condition, and batch effect still exists for certain genes after normalization of the data [19].

#### Nitric oxide

This study was designed to investigate whether exposing mammalian cells to nitric oxide (NO) stabilizes mRNAs [10]. Human lung fibroblast cells (IMR90) were exposed to NO for 1 h, then transcription inhibited together with control cells for 7.5 h. Expressions in the exposed sample and control cells are measured at 0 h and 7.5 h using Affymetrix HG-U133A microarray, resulting in 4 arrays for each cell pair. The experiment was repeated at 3 different times. The dataset contains the 3 batches of data, each containing 4 arrays of different treatment combinations, which leads to 12 samples in total.

#### Oncogenic signature

The growth factor receptor network (GFRN) contributes to breast cancer progression and drug response. This RNA-Seq dataset is designed to develop gene signatures for several GFRN pathways: AKT, BAD, HER2, IGF1R, RAF1, KRAS, and EGFR. We used recombinant adenoviruses to express these genes in case samples and produce green fluorescent protein (GFP) in control samples, using replicates of human mammary epithelial cells (HMECs). RNA-Seq data are collected from these HMECs overexpressing GFRN genes and GFP controls [21]. This dataset contains 89 samples, which are created in three batches: batch 1 contains 6 replicate samples of each for AKT, BAD, IGF1R, and RAF1, 5 replicates for HER2, and 12 replicates for GFP controls (GEO accession GSE83083); batch 2 consists of 9 replicates of each for three types of KRAS mutants and GFP control (GEO accession GSE83083); batch 3 contains 6 replicates of each for EGFR and its corresponding control (GEO accession GSE59765). We derived signatures from this dataset and predicted pathway activities and drug effects in cell line and patient datasets with ASSIGN [23].

#### Lung cancer

This dataset contains microarray measurements from histologically normal bronchial epithelium cells collected during bronchoscopy from non-smokers, former smokers, and current smokers. Samples are selected from various studies, which are divided into three batches A (GSE994 [24], GSE4115 [25, 26], GSE7895 [27]), B (GSE66499, [28]) and C (GSE37147, [29]). The three sub-batches within A are ComBat adjusted before A is combined with B and C. The dataset contains 1051 samples, with 318 samples in batch A, 507 in batch B, and 226 in batch C.

## Results

### Moments-based tests of significance for batch effect

We introduced sample- and gene-wise tests to detect significant differences in the moments of batch effect distributions. We applied these tests to four different datasets (Table 1) and observed their properties. We found that the four datasets have different degrees of batch effect, and require different adjustment. The first dataset (bladder cancer) has significant mean differences between the batches (*P*<0.0001), but has *P*-values above 0.33 for variance differences for both tests. Since the bladder cancer dataset only exhibits batch effects in the mean and not in the variance, mean-only adjustment is more suitable for this dataset. In the nitric oxide dataset, however, mean/variance ComBat is required to remove the difference in batch variances detected by the gene-wise test (*P*=0.0005 without adjustment; *P*=0.0042 using mean-only ComBat). All four datasets show certain levels of significant differences in skewness and/or kurtosis even after the mean/variance ComBat is used, which suggests that adjustment for higher order moments may be required, which is beyond the scope of this paper.

**Table 1:** *P*-values from sample-wise and gene-wise *robust* tests on four datasets, before and after batch correction

		Sample-wise tests				Gene-wise tests			
Dataset	ComBat	Mean	Variance	Skewness	Kurtosis	Mean	Variance	Skewness	Kurtosis
Bladder cancer	None	<0.0001	0.6495	0.0539	0.3149	<0.0001	0.3353	<0.0001	0.0012
	Mean-only	0.9998	0.9557	0.1496	0.6236	0.2011	0.3618	<0.0001	0.0012
	Mean/variance	1	0.8989	0.1826	0.2737	0.2538	0.9816	<0.0001	0.0012
Nitric oxide	None	0.1007	0.3565	0.1009	0.866	<0.0001	0.0005	<0.0001	0.9887
	Mean-only	0.9997	0.577	0.9838	0.9485	0.4595	0.0042	<0.0001	0.9887
	Mean/variance	1	0.982	0.9847	0.7013	0.7245	0.6219	<0.0001	0.9791
Oncogenic signature	None	0.0011	<0.0001	0.0001	0.0235	<0.0001	0.0001	<0.0001	0.5711
	Mean/variance	1	0.7486	0.5553	0.9202	0.0363	0.8919	<0.0001	0.5711
Lung cancer	None	<0.0001	<0.0001	<0.0001	<0.0001	<0.0001	0.0106	<0.0001	0.4853
	Mean/variance	1	0.9872	0.0003	0.9612	0.0016	0.9971	<0.0001	0.4853

The four datasets have different degrees of batch effect. The bladder cancer dataset has differences in batch mean, but does not show any batch effect in the variance. Mean-only ComBat is sufficient to adjust this dataset as there is no need to adjust the variance. In the nitric oxide dataset, the gene-wise test reports significant differences in both the mean and the variance. The full mean/variance ComBat is necessary to remove batch effects in this data. The mean/variance ComBat cannot adjust the skewness or kurtosis. All four datasets exhibit certain levels of batch effect in the skewness and/or kurtosis, which may call for methods that adjust these higher order moments. Results comparing robust and non-robust F tests are summarized in Additional file 3

### Mean-only batch adjustment

We modified the current mean/variance ComBat into a mean-only version of ComBat, which allows users to only adjust the batch effects in mean. It is recommended for cases where milder batch effects are expected (i.e. there is no need to adjust the variance). For example, we have shown in Table 1 and Additional file 3 that in the bladder cancer dataset, the mean, skewness and kurtosis are significantly different across batches. But there is no evidence for significant differences in the variance.

**Fig. 1:**
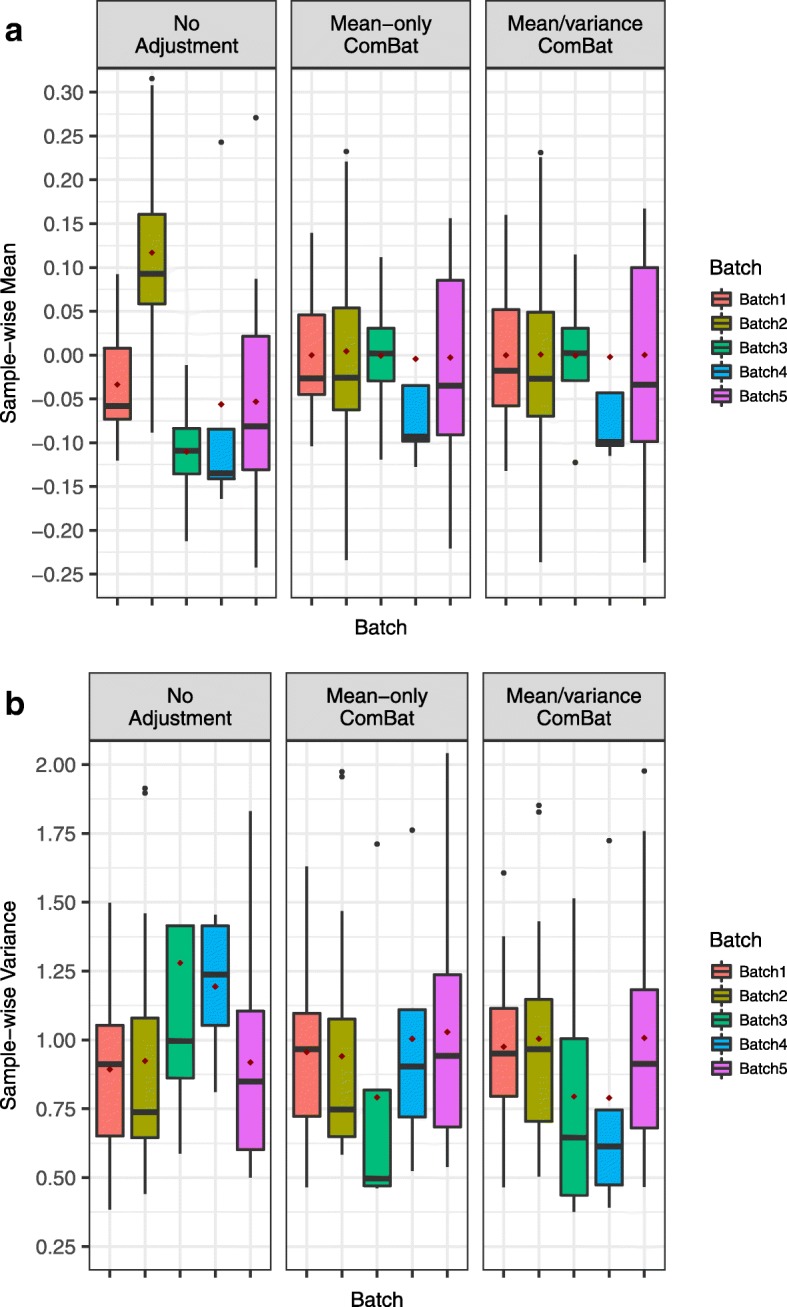
Distribution of sample-wise mean and variance estimates from each batch in the bladder cancer data. Estimates are calculated within each sample as previously described. **a** Boxplots of sample-wise mean estimates ($\bar {\gamma }_{ij}$, as in Eq. (3)) within each batch. The sample-wise mean estimates for batch 2 in the unadjusted data are significantly different from the other batches. Both mean-only and mean/variance ComBat adequately correct this batch 2 mean difference. **b** Boxplots of sample-wise variance estimates across batches. The sample-wise variance estimates are not significantly different in the unadjusted data. Adjusting either just the mean or both mean and variance does not makes the estimates more similarly distributed, meaning that adjusting the variance is not necessary

We applied both the mean-only and mean/variance ComBat on the bladder cancer dataset to compare their performances. We compared batch mean ($\bar {\gamma }_{ij}$ from Eq. (3)) and variance estimates collected within each sample in the unadjusted data, and in data adjusted by the two versions of ComBat (Fig. 1). Consistent with the result in Table 1 (*P*<0.0001), the mean estimates in the original data are significantly different across batches. In particular, Fig. 1a shows mean-level differences in batch 2 compared to the other batches. Because variance estimates are not significantly different across batches, mean-only ComBat is sufficient to adjust the bladder cancer data. Neither version of ComBat makes the variance estimates more similarly distributed to each other than they are in the unadjusted data (Fig. 1b). This shows that, based on the sample-wise test, adjusting both the mean and variance of batch effects in the bladder cancer data does not give better results than only adjusting the mean.

**Fig. 2:**
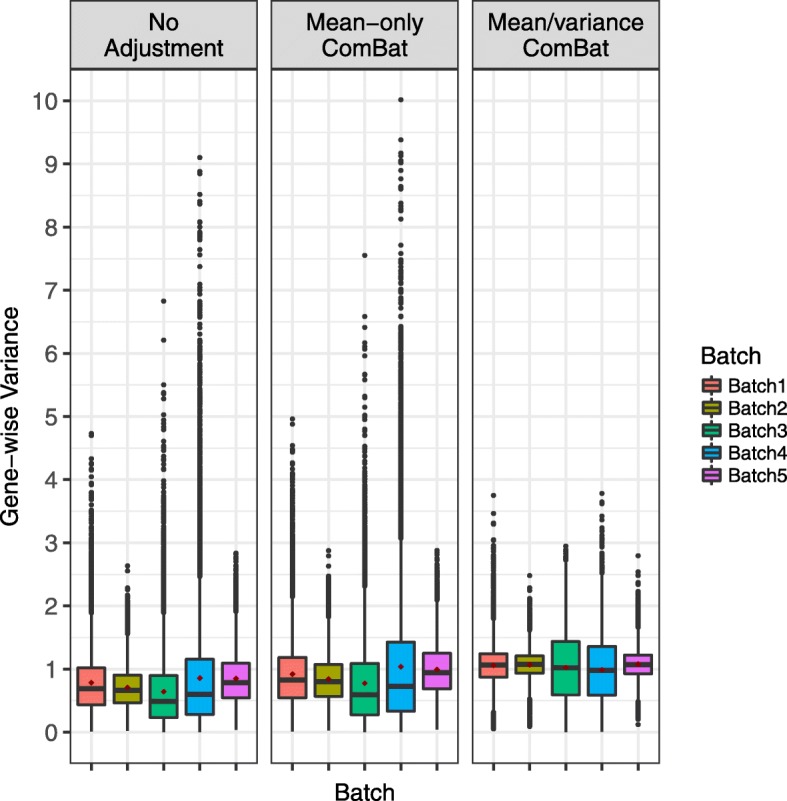
Distribution of gene-wise variance estimates from each batch in the bladder cancer data. Batch 3 and batch 4 have smaller sample size than the other batches, thus their variance estimates are impacted more by outlying samples. Mean/variance ComBat brings all estimates to the same levels, over correcting the variance estimates in batches 3 and 4. This leads to unwanted, less variable gene expression (see Additional file 1: Figure S1). Mean-only ComBat does not affect or overcorrect the variance estimates

From the gene-wise perspective, we found that the mean/variance ComBat overcorrects the data by shrinking the variance of batch 3 and 4 when pooling information from all batches (Fig. 2, Additional file 1: Figure S1). Batch 3 (4 samples) and batch 4 (5 samples) have relatively fewer samples than the remaining three batches (11, 18 and 19 samples), and so the gene-wise variance estimates are more likely to be impacted by outlying samples. When mean/variance ComBat estimates the background variance using all batches, variance estimates become less variable in these two batches than in the other batches. Thus, the variance adjustment actually introduces differences in distribution across batches than in the original data (Additional file 1: Figure S1). In contrast, mean-only ComBat does not affect the variance estimates, thus avoiding the overcorrection problem. Therefore, mean-only ComBat is more justifiable than mean/variance ComBat for the bladder cancer data, where there is no need to adjust the variance.

#### Selecting the appropriate ComBat version for each dataset

Unlike in the bladder cancer data, mean-only ComBat is not sufficient for removing batch effects in the other three datasets. For example, the oncogenic signature dataset displays batch effect in both mean and variance. Mean/variance adjustment is required to remove the technical differences across batches (Additional file 1: Figure S2). As another example, the gene-wise test detects a significant difference in variance (Table 1, *P*=0.0005) in the nitric oxide dataset. In this dataset, we found that mean-only ComBat cannot completely remove the significant difference in gene-wise variance estimates across batches (Additional file 1: Figure S3). In this case, the mean/variance ComBat is necessary to remove the batch effect.

We emphasize that it is critical to select the appropriate ComBat version based on the degree of batch effect in different datasets. We simulated datasets with two condition groups, with some genes that differentially express between the two groups. Samples are divided in two batches. We simulated three types of batch effect in the data: 1) no batch effect, 2) only differences in the mean, and 3) both mean and variance batch effects. We applied both the mean-only and the mean-variance ComBat on each dataset. Then in both adjusted and unadjusted data, we performed differential expression analysis, and calculated the type I error rate and statistical power of our detection. We observed that using the ComBat model corresponding to the type of batch effect in the data is able to gain more power of detection, at the same cost of type I error rate increase (Additional file 1: Figure S4). These results show that a mean-variance model overfits the data in cases where a mean-only adjustment is needed, and that the mean-only model is not always sufficient. Therefore, it is necessary to evaluate the degree of batch effect, and select the appropriate ComBat version for batch correction. More details of this analysis are available in Additional file 1.

We also note that the nitric oxide dataset gives conflicting results for the sample-wise and gene-wise variance tests. We emphasize that, though both the sample- and gene-wise tests intend to detect differences in the hyper-moments across batches, they interrogate different aspects of the batch effect: sample-wise *P*-values reflect the difference in moments between batches by summarizing information over genes; while gene-wise *P*-values neglect differences between samples by summarizing across samples to estimate the gene-wise moments. We have shown in our previous work that multiple diagnostics are often needed to fully diagnose batch effects, as batch effects can be present in many different ways [20]. Thus we recommend using mean/variance ComBat if either of the gene-wise or sample-wise tests show a significant batch effect.

### Higher order moment-based batch adjustment

**Fig. 3:**
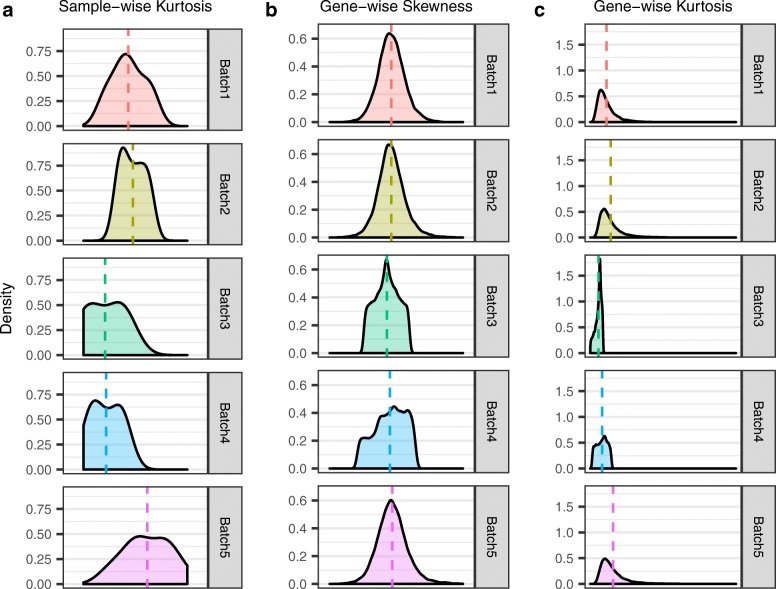
Distributions of higher order moments in the bladder cancer dataset after the mean/variance adjustment. The current mean/variance ComBat does not adjust higher order moments, thus distributions of these moment estimates remain significantly different (**a** sample-wise kurtosis: *P*=3.025*e*−05 using *non-robust* test; **b** gene-wise skewness: *P*=0; **c** gene-wise kurtosis: *P*=0.0012 using *robust* test) across batches even after batch adjustment. These may cause problems in downstream analysis such as prediction tasks, and call for batch correction methods that adjust the higher order moments

We observed evidence in all four datasets that the current ComBat mean/variance model does not completely remove all batch effects (Fig. 3). The bladder cancer has significant differences in gene-wise kurtosis even after mean/variance adjustment. The lung cancer data has remaining batch effect in sample-wise skewness. Also, the gene-wise test on skewness remains significant in all datasets (Table 1). These results suggest that a more severe batch correction targeting the higher order moments may be necessary, indicating the need to develop additional methods and tools for these cases.

### Batch adjustment based on a reference batch

We used pathway signature projection examples to establish the benefits of reference-batch ComBat. First, we use a simulated pathway dataset to compare the benefits of the original and new reference-batch versions of ComBat. The goal of this simulation is to justify the necessity of reference-batch ComBat in scenarios when one batch is of superior quality than the other batches, or when biomarkers need to be generated in one dataset, fixed, and then applied to another dataset. We further illustrate that reference-batch ComBat yields better prediction results than the original ComBat in a real data signature profiling example for predicting drug efficacy.

#### Simulation study

We used simulated data to represent a gene expression signature study for an activated (or knocked down) biological pathway or drug perturbation that is profiled into another batch of data to predict pathway activity (or drug efficacy). Descriptions of these simulated datasets are detailed in the Dataset descriptions - Pathway simulation section. We used the two versions of ComBat (original and reference) to combine the two batches and to enable the prediction of the activity strength of the pathway from batch 1 into the batch 2 samples. Batch 1 was selected as reference for the reference-batch ComBat. Pathway activation levels are added in both versions ComBat as covariates. Results of not using activation levels as covariates is shown in Additional file 1: Figure S5.

**Fig. 4:**
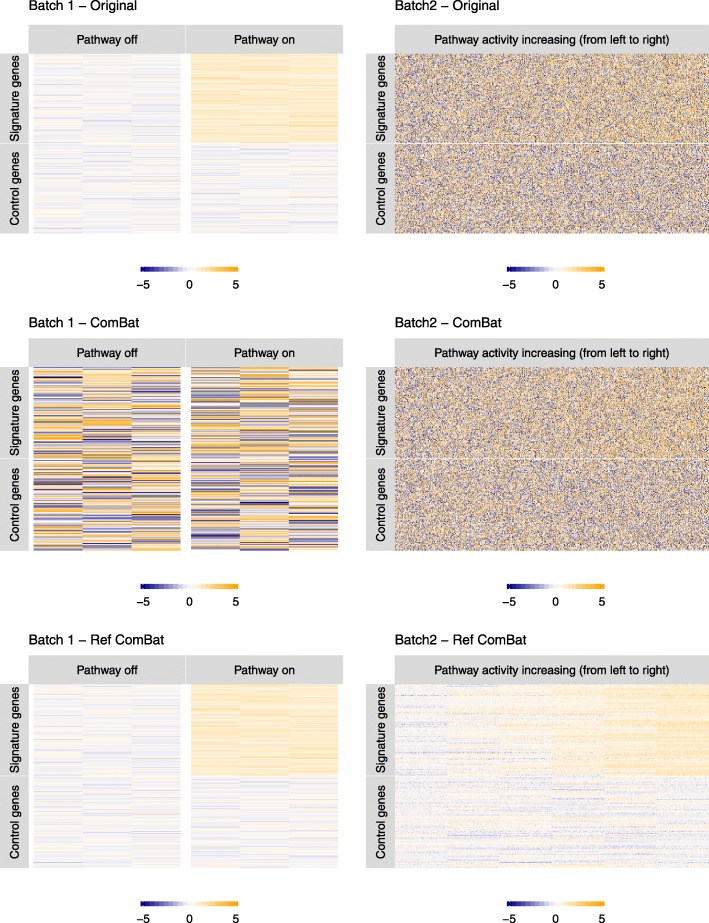
Simulated pathway datasets before and after batch correction using original and reference-batch ComBat. The figure shows the heatmaps of the gene-by-sample expression matrices for the two simulated batches. Pathway activation levels are included as covariates in the two versions of ComBat. Batch 1 is less variable than batch 2, and is better in quality for identifying signatures for the pathway. Using the original ComBat does not remove the variance in batch 2. Instead, it causes a severe loss of signal in batch 1 by inflating the variance. Reference-batch ComBat does not change the chosen reference (batch 1) and leads to clearer signal detection in batch 2

**Fig. 5:**
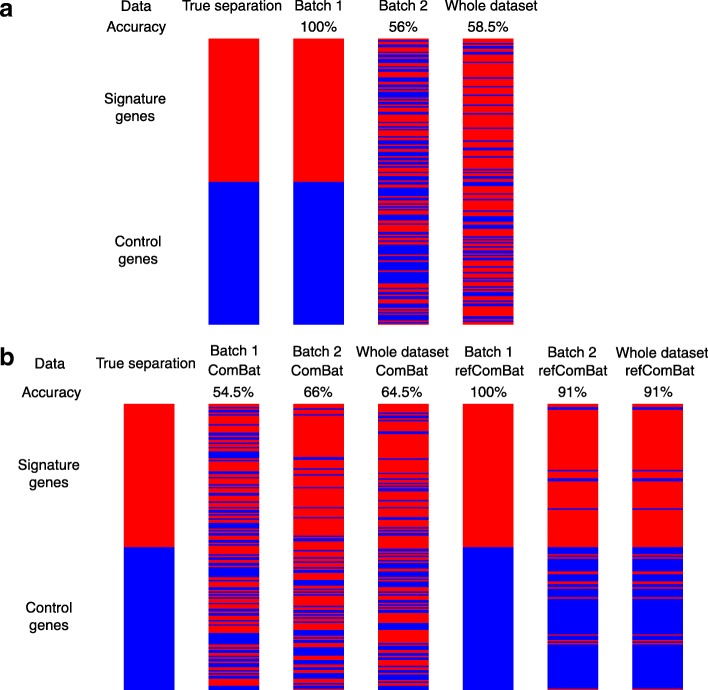
Cluster assignment of the 200 genes using k-means algorithm, where k=2. Color bars show the 200 genes from top to bottom, which corresponds to the gene labels in Fig. 4. The red and blue bars represent signature and control genes, respectively. During batch adjustment, true activation levels are included as covariates, as opposed to using no covariates in both versions of ComBat (Additional file 1: Figure S6). In the batch adjusted data, we first clustered genes into 2 groups without specifying the group sizes or labels. Then, clusters are assigned as signature and control by how it best accords with the original separation. **a** In batch 1, genes are correctly separated. But combining batch 2 with batch 1 without ComBat adjustment changes the signature / non-signature separation. Only 58.5% genes remain the same in the combined dataset. **b** Reference-batch ComBat gives cluster assignment that is more consistent with the true separation than original ComBat, in batch 1 only, batch 2 only, and the combined dataset of batch 1 and 2. These results suggest that the original ComBat breaks the similarity between genes in the same group (signature or control), where similarity is measured by the Euclidean distance. Only reference-batch ComBat is able to preserve this similarity

The original and reference-batch ComBat yield very different results in the two batches (Fig. 4). The original ComBat uses the overall mean and variance of each gene across all batches as a background profile. Due to the large sample size and variances of the second batch, the estimated background profiles resemble batch 2 in variance. As a result, ComBat significantly increased the variance of batch 1 to match the variance of batch 2. As illustrated in Fig 4, the original ComBat results in a near complete loss of signal in batch 1. In comparison, reference-batch ComBat does not change the chosen reference (batch 1). It estimates the background means and variances based on batch 1, and adjusts batch 2 accordingly. After adjustment, the true signals of the pathway are recovered in the second batch. In this setting where batch 1 is of better quality, but batch 2 is more variable and larger in size, reference-batch ComBat retrieves biological signals of interest more successfully than the original version. This is further demonstrated quantitatively by the k-means clustering shown in Fig. 5. However, we note that the true activation level of signature genes are included as covariates in ComBat in this example. In a more realistic setting, the activation levels are unknown and cannot be included as covariates in the ComBat adjustment. When we applied ComBat without covariates (Additional file 1: Figure S5), the pathway activation signals are less clear in both batches. However, the original version of ComBat still increases the variance in batch 1, making the data less ideal for signature development than those from the reference version.

To quantify the impact of batch correction on batch 1, we use a k-means clustering approach to attempt to identify the biomarker gene set (the first 100 genes are the signature genes and the subsequent 100 genes are unaffected by the perturbation). We treat the gene expression of each sample as high-dimensional vectors (batch 1: 6 samples ; batch 2: 600 samples). We used k-means clustering to divide these vectors into two groups for batch 1 alone, batch 2 alone, and batches 1 and 2 combined, with both ComBat adjustments (original and reference). We compared the clustering assignment of genes with the signature/non-signature separation, and calculated the accuracy as the maximum percentage of correctly classified genes in either way of labeling the two clusters as signatures and non-signatures. We evaluated how using original and reference-batch ComBat affects this accuracy.

In batch 1 without adjustment, all genes are correctly separated into signature and non-signature. However, this separation is confounded when batch 2 is combined with batch 1, as only 58.5% of the genes are correctly separated in the combined dataset. When using original ComBat, because the variance of batch 1 is artificially increased, the accuracy in batch 1 alone drops from 100 to 54.5%, and only 64.5% of the genes maintain their correct signature/non-signature labels after combining batch 2 with batch 1. In contrast, reference-batch ComBat keeps the cluster assignment in the adjusted batch 1 100% correct, because batch 1 stays intact as the reference, and 91% of the genes retain their correct labels in the combined dataset after adjustment. Thus reference ComBat improves the ability to identify biomarker genes across multiple studies compared to no adjustment and standard ComBat.

#### EGFR signature and drug prediction

We also considered a real signature study using ASSIGN [23], a pathway profiling toolkit based on a Bayesian factor analysis approach, to develop an EGFR pathway signature from the oncogenic signature dataset. ASSIGN allows for derivation of signatures from a pathway perturbation experiment, and adapts signatures from experimental datasets to disease. Our goal was to predict EGFR pathway activity in two RNA-Seq datasets: a breast cancer cell line panel [30] and from breast carcinoma patients in TCGA [31]. As in the simulation study, the two RNA-Seq test sets were first combined with the EGFR training set separately, to adjust for the batch effect between the training and the test set. ASSIGN then trains biomarkers from the adjusted EGFR training set, and makes predictions of pathway activity in both of the adjusted test sets. We compared the impact of using three versions of ComBat (original, mean-only and reference-batch), as well as frozen SVA and RUV on these predictions (Table 2).

**Table 2:** Comparison between five batch correction methods in predicting pathway activity and drug efficacy

		Correlation: EGFR protein expression		Drug response in cell lines	
ComBat version	Common genes (cell line vs. TCGA)	Cell line	TCGA	Erlotinib	GSK1120212
Original ComBat	20 (40%)	0.316	0.132	0.360	0.401
Mean-only ComBat	44 (88%)	0.331	-0.042	0.294	0.407
Reference-batch ComBat	50 (100%)	0.442	0.299	0.415	0.520
Frozen SVA	50 (100%)	0.115	0.092	-0.09	-0.131
RUV	40 (80%)	0.287	0.182	0.332	0.145

We combined the oncogenic signature dataset with the cell line and TCGA patient data separately to adjust for batch effect and enable the profiling of EGFR signatures from the oncogenic data to the test sets. We observed the set bias using original ComBat (40% same signature genes), mean-only ComBat (88% same genes), and RUV (80% same genes) to combine the datasets. Reference-batch ComBat and frozen SVA kept the same signature genes. Also, using reference-batch ComBat gave the highest correlations of prediction scores with both protein expression and drug response, among all five batch correction methods. These results support the benefit of using reference-batch ComBat in this context

We used ASSIGN to develop a 50-gene signature from the EGFR samples in the training set [21]. We first focus on the three versions of ComBat in the ability to generate replicable signatures. Because of the ’set bias’ caused by using original ComBat, only 20 (40%) of the signature genes are the same between the signatures developed in the training set adjusted against the cell line test set compared to the training set adjusted against the patient data. The same analysis performed with mean-only ComBat produced gene signatures with 44 (88%) of the genes shared between the two datasets. Because reference-batch ComBat does not change the EGFR dataset, the signatures are identical after (separate) adjustment with the two test sets. This points to the value of using reference ComBat to develop fixed genomic biomarkers that can be projected into multiple datasets, even at different times and without the need to combine all the data together.

We further compared the correlations of pathway predictions with the following validation datasets: (1) EGFR protein expression data (cell line and TCGA), and (2) EGFR inhibitor drug response (cell lines). As shown in Table 2, the correlations with protein expression for the reference-batch ComBat adjusted data (Cell Line: 0.442, TCGA: 0.299) are the highest in both test sets among all five methods. The correlations with drug response are also the highest when adjusting the data with reference-batch ComBat. For example, reference adjusted data yield a correlation of 0.415 with Erlotinib response and 0.520 with GSK1120212 response, compared to using the original (Erlotinib: 0.360, GSK: 0.401) and mean-only (Erlotinib: 0.294, GSK: 0.407) ComBat, frozen SVA (Erlotinib: -0.09, GSK: -0.131), and RUV (Erlotinib: 0.332, GSK: 0.145). These results strongly justify the benefits of the reference version of ComBat in pathway profiling and predicting drug efficacy.

## Discussion

Combining multiple genomic datasets is beneficial for boosting statistical power of studies, especially in cases where data are generated in small batches necessitated by high experimental cost or difficulties in collecting samples. In addition, combining batches of data from similar experiments from different labs also provides opportunities for increased power for the detection of biological differences, as well as providing ways for testing/validating biomarkers generated in one batch of data. The presence of batch effects due to technical heterogeneity and batch effects due to different profiling platforms, protocols, or other factors can often confound the biological signals in data, which reduces the benefit of combining datasets. Despite the many previously developed techniques for batch adjustment, there are still situations where new methods need to be developed to appropriately or more effectively remove batch effects.

We introduced new models and tools for addressing batch correction in several scenarios based on the ComBat model. For example, many methods focus on adjusting the means and variances of batches, but some datasets require less (or more) stringent adjustments. We proposed two significance testing approaches, based on the batch effect moment distributions, to diagnose the degree of adjustment required. We visualized different degrees of batch effect detected by these tests in four experimental datasets. In the bladder cancer data where mean is significantly different across batches but not the variance, our proposed mean-only ComBat successfully removes the batch differences. In all four datasets, we presented evidence that adjusting both mean and variance is not sufficient to remove all batch effect, which calls for a method that performs a more severe batch adjustment.

For datasets where a less severe adjustment targeting the mean is sufficient, adjusting the variance may lead to unnecessary costs in downstream analysis. The original ComBat model pools all samples to estimate both the mean and the variance batch effects, which introduces a correlation structure between the samples. Such correlations may cause issues in further analysis, such as inflating type I error rate in differential expression detection, if we do not account for them properly. Therefore, for the datasets with only mean batch effect, our proposed mean-only ComBat is able to avoid the cost of estimating the variances, and is more beneficial than the mean-variance version.

It is important to highlight that a thorough evaluation of the degree of batch effect is necessary before applying any version of ComBat. We presented simulation results to demonstrate that using the ComBat model corresponding to the type of batch effect in the data is able to achieve more statistical power at the same cost of type I error rate in differential expression analysis. Therefore, we strongly suggest evaluating whether there is differences in mean and variances between batches with the moment-based significance tests, and selecting the appropriate version of ComBat based on the type of batch effect in the data.

Also, we noticed that the gene-wise and sample-wise tests yielded different results in a few of our datasets. To resolve a conflicting result from the two tests, we recommend visualizing the batch distributions as we did in this study, and decide the level of adjustment required based on all available information. BatchQC offers visualizations from various aspects including boxplot of gene expressions, dendrogram of clustering, and scatter plot of principal components, which can all assist in diagnosing the degree of batch effect.

In addition, we illustrated the benefits of selecting a reference batch in batch correction, in situations when one batch is high quality and less variable, and when biomarkers need to be developed from one study, fixed and validated on another study. Particularly in the situation where the goal is to generate fixed biomarkers, including an extra batch of data to those in hand can strongly affect the results, an issue described as ‘set bias’. In these situations, analysis need to be re-run in order to process the new batch of data, which can cause the biomarker genes to be largely different.

Our reference-batch ComBat is proven more successful in retrieving biological signals in signature profiling examples, where one batch shows a clear signal of biological conditions. We demonstrated that reference-batch ComBat resolves the ‘set bias’ caused by the original version of ComBat in adding data sequentially, and yields better prediction of pathway activities and drug effects. Although these approaches are only alternative expressions of the ComBat model, their implementation has critical impact in real batch correction scenarios.

## Conclusions

We proposed diagnostic tools and improved models based on ComBat to evaluate and address batch effects in certain batch adjustment situations. The significance tests for batch differences can be used to determine the degree of batch effects to be adjusted. We purposed mean-only ComBat for the situation where a less severe adjustment is preferred. The reference-batch ComBat is able to leave one batch unchanged, which is especially useful for generating a fixed biomarker for further clinical use. We have shown in both simulations and real data that these proposed methods provide better solutions to batch effects than the existing ComBat model in their corresponding batch correction scenarios.

## Additional files

Additional file 1 Supplementary materials (PDF 1612 kb)

Additional file 2 Mean and variance of gene expression distributions estimated from the EGFR signature and the TCGA breast cancer patient datasets. In TCGA, we used proteomics data of the patients, and binned the EGFR protein expression into 6 gradually increasing levels, partitioning all patients into 6 equal-sized groups. Mean and variances are estimated within each group. Up- and down-regulated genes are both EGFR signature genes derived by ASSIGN. The design and parameters for our simulation studies resemble the real estimates in these tables. Batch 1 represents the EGFR signature dataset with small gene variances, and a clear separation between the two condition groups in the expression of up-regulated genes. Batch 2 resembles the TCGA patient data with much larger variances than Batch 1. (XLSX 10 kb)

Additional file 3 Comparison of *P*-values for applying robust and non-robust F tests on the four experimental datasets. (XLSX 12 kb)

## Abbreviations

CIS: carcinoma in situ
DWD: distance weighted discrimination
GEO: the Gene Expression Omnibus database
GFP: green fluorescent protein
GFRN: growth factor receptor network
HMEC: human mammary epithelial cell
NO: nitric oxide
RUV: remove unwanted variation
sTCC: superficial transitional cell carcinoma
SVA: surrogate variable analysis
SVD: singular value decomposition
TCGA: The Cancer Genome Atlas
XPN: cross-platform normalization

## References

[1] Soon WW, Hariharan M, Snyder MP (2013). High-throughput sequencing for biology and medicine. Mol Syst Biol.

[2] Reuter JA, Spacek DV, Snyder MP (2015). High-throughput sequencing technologies. Mol Cell.

[3] Van Dijk EL, Auger H, Jaszczyszyn Y, Thermes C (2014). Ten years of next-generation sequencing technology. Trends Genet.

[4] Tomczak K, Czerwińska P, Wiznerowicz M (2015). The cancer genome atlas (tcga): an immeasurable source of knowledge. Contemp Oncol.

[5] Kupfer P, Guthke R, Pohlers D, Huber R, Koczan D, Kinne RW (2012). Batch correction of microarray data substantially improves the identification of genes differentially expressed in rheumatoid arthritis and osteoarthritis. BMC Med Genom.

[6] Luo J, Schumacher M, Scherer A, Sanoudou D, Megherbi D, Davison T, Shi T, Tong W, Shi L, Hong H (2010). A comparison of batch effect removal methods for enhancement of prediction performance using maqc-ii microarray gene expression data. Pharmacogenomics J.

[7] Alter O, Brown PO, Botstein D (2000). Singular value decomposition for genome-wide expression data processing and modeling. Proc Natl Acad Sci.

[8] Benito M, Parker J, Du Q, Wu J, Xiang D, Perou CM, Marron JS (2004). Adjustment of systematic microarray data biases. Bioinformatics.

[9] Shabalin AA, Tjelmeland H, Fan C, Perou CM, Nobel AB (2008). Merging two gene-expression studies via cross-platform normalization. Bioinformatics.

[10] Johnson WE, Li C, Rabinovic A (2007). Adjusting batch effects in microarray expression data using empirical bayes methods. Biostatistics.

[11] Gagnon-Bartsch JA, Speed TP (2012). Using control genes to correct for unwanted variation in microarray data. Biostatistics.

[12] Leek JT, Johnson WE, Parker HS, Jaffe AE, Storey JD (2012). The sva package for removing batch effects and other unwanted variation in high-throughput experiments. Bioinformatics.

[13] Patil P, Bachant-Winner P-O, Haibe-Kains B, Leek JT (2015). Test set bias affects reproducibility of gene signatures. Bioinformatics.

[14] Lazar C, Meganck S, Taminau J, Steenhoff D, Coletta A, Molter C, Weiss-Solís DY, Duque R, Bersini H, Nowé A (2012). Batch effect removal methods for microarray gene expression data integration: a survey. Brief Bioinform.

[15] Kitchen RR, Sabine VS, Sims AH, Macaskill EJ, Renshaw L, Thomas JS, van Hemert JI, Dixon JM, Bartlett JM (2010). Correcting for intra-experiment variation in illumina beadchip data is necessary to generate robust gene-expression profiles. BMC Genom.

[16] Sîrbu A, Ruskin HJ, Crane M (2010). Cross-platform microarray data normalisation for regulatory network inference. PLoS ONE.

[17] Bolstad BM, Irizarry RA, Åstrand M, Speed TP. A comparison of normalization methods for high density oligonucleotide array data based on variance and bias. Bioinformatics. 2003;19(2):185–93. 10.1093/bioinformatics/19.2.185 12538238

[18] Lin M, Lucas Jr HC, Shmueli G (2013). Research commentary—too big to fail: large samples and the *p*-value problem. Inf Syst Res.

[19] Leek JT, Scharpf RB, Bravo HC, Simcha D, Langmead B, Johnson WE, Geman D, Baggerly K, Irizarry RA (2010). Tackling the widespread and critical impact of batch effects in high-throughput data. Nat Rev Genet.

[20] Manimaran S, Selby HM, Okrah K, Ruberman C, Leek JT, Quackenbush J, Haibe-Kains B, Bravo HC, Johnson WE (2016). Batchqc: interactive software for evaluating sample and batch effects in genomic data. Bioinformatics.

[21] Rahman M, MacNeil SM, Jenkins DF, Shrestha G, Wyatt SR, McQuerry JA, Piccolo SR, Heiser LM, Gray JW, Johnson WE (2017). Activity of distinct growth factor receptor network components in breast tumors uncovers two biologically relevant subtypes. Genome Med.

[22] Leek JT. bladderbatch: Bladder gene expression data illustrating batch effects. R package version 1.18.0. 2018. Available at https://www.bioconductor.org/packages/release/data/experiment/html/bladderbatch.html. Accessed 30 June 2018.

[23] Shen Y, Rahman M, Piccolo SR, Gusenleitner D, El-Chaar NN, Cheng L, Monti S, Bild AH, Johnson WE (2015). Assign: context-specific genomic profiling of multiple heterogeneous biological pathways. Bioinformatics.

[24] Spira A, Beane J, Shah V, Liu G, Schembri F, Yang X, Palma J, Brody JS (2004). Effects of cigarette smoke on the human airway epithelial cell transcriptome. Proc Natl Acad Sci U S A.

[25] Spira A, Beane JE, Shah V, Steiling K, Liu G, Schembri F, Gilman S, Dumas Y-M, Calner P, Sebastiani P (2007). Airway epithelial gene expression in the diagnostic evaluation of smokers with suspect lung cancer. Nat Med.

[26] Gustafson AM, Soldi R, Anderlind C, Scholand MB, Qian J, Zhang X, Cooper K, Walker D, McWilliams A, Liu G (2010). Airway pi3k pathway activation is an early and reversible event in lung cancer development. Sci Transl Med.

[27] Beane J, Sebastiani P, Liu G, Brody JS, Lenburg ME, Spira A (2007). Reversible and permanent effects of tobacco smoke exposure on airway epithelial gene expression. Genome Biol.

[28] Silvestri GA, Vachani A, Whitney D, Elashoff M, Porta Smith K, Ferguson JS, Parsons E, Mitra N, Brody J, Lenburg ME (2015). A bronchial genomic classifier for the diagnostic evaluation of lung cancer. N Engl J Med.

[29] Steiling K, Van Den Berge M, Hijazi K, Florido R, Campbell J, Liu G, Xiao J, Zhang X, Duclos G, Drizik E (2013). A dynamic bronchial airway gene expression signature of chronic obstructive pulmonary disease and lung function impairment. Am J Respir Crit Care Med.

[30] Daemen A, Griffith OL, Heiser LM, Wang NJ, Enache OM, Sanborn Z, Pepin F, Durinck S, Korkola JE, Griffith M (2013). Modeling precision treatment of breast cancer. Genome Biol.

[31] Network CGA (2012). Comprehensive molecular portraits of human breast tumours. Nature.

[32] Edgar R, Domrachev M, Lash AE (2002). Gene expression omnibus: Ncbi gene expression and hybridization array data repository. Nucleic Acids Res.

