# Supplementary material for: Alternative empirical Bayes models for adjusting for batch effects in genomic studies
Source: BMC Bioinformatics. 2018 Jul 13;19:262. doi: 10.1186/s12859-018-2263-6 (PMC6044013; doi:10.1186/s12859-018-2263-6)
Supplement: Supplementary file 1 — Supplementary materials (PDF 1612 kb) [file 12859_2018_2263_MOESM1_ESM.pdf]

Supplementary Materials for "Alternative empirical Bayes models for adjusting for batch effects in genomic studies"

Yuqing Zhang, David F. Jenkins, Solaiappan Manimaran, W. Evan Johnson

F statistics for robust sample- and gene-wise tests

The robust F-test is conducted similarly to the standard F-test by comparing two nested models. Then the test is made robust by adding a variance inflation factor Δ to the standard F-statistics:

$$F_R = \frac{Explained\ variance}{\Delta + Unexplained\ variance}$$

For example, in the gene-wise test, two linear regression models are applied to the moment estimate values $\bar{\gamma}_{ig}$ (separately for the mean, variance, skewness, and kurtosis): the full model contains batch as a predictive factor, whereas the reduced model contains only a constant intercept. The robust F-statistic is computed as follows with a suitably selected Δ:

$$F_R = \frac{(RSS_0 - RSS_1)/(df_1 - df_0)}{\Delta + (RSS_1/(n - df_1))}$$
$$\Delta = n * (mean_{ig}(\bar{\gamma}_{ig}) * 0.05)^2$$

RSS_0: Residual Sum of Squares for the reduced model; RSS_1: Residual Sum of Squares for the full model; $df_1 = \#$ batch; $df_0 = 1$; n= # genes * # batch

The Δ in the equation is selected heuristically as the total degrees of freedom times the squared 5% of the average moment estimates. This variance inflation factor reduces the statistical significance when the effect size of the batch is much smaller than the expression level of the normalized data.

Selecting the appropriate ComBat model for different datasets

We simulated a differential expression task with different types of batch effect in the data, to demonstrate the necessity to select the appropriate ComBat model based on the data. We generated datasets containing 2 batches and 2 condition (case/control) groups. In each simulation, we generated 1) a dataset with no batch effect between the two batches, 2) a dataset with only differences in the batch mean, and 3) a dataset with both mean and variance batch effects between the two batches. We generated gene expression from Normal distributions, such that 100 out of 10000 simulated genes are upregulated between the two condition groups. We used mean and variance parameters that are arbitrarily selected, and estimated from the real nitric oxide dataset for the Normal distributions. We then applied both the mean-only and the mean-variance adjustments to each of the three datasets. In both the adjusted and unadjusted data, we used limma to detect differentially expressed genes between condition groups. Finally, we calculated the type I error rate and the statistical power of our predictions.

We quantified the performance of the mean-only and the mean-variance ComBat with the following trade-off score:

$$\frac{Power_{adjusted} - Power_{unadjusted}}{Type\ I\ error\ rate_{adjusted} - Type\ I\ error\ rate_{unadjusted}} \tag{1}$$

At the same cost of type I error rate increase, a better performing batch adjustment model will give a larger increase in the power for detecting differentially expressed genes. Therefore, the better model will have a larger trade-off score as defined by equation (1). We repeated the simulation for 100 times, and visualized the distribution of the trade-off scores in each batch effect type / batch adjustment model combination (Figure S4). These results show that using the model corresponding to the type of batch effect in the data yields better trade-off of statistical power over type I error rate.

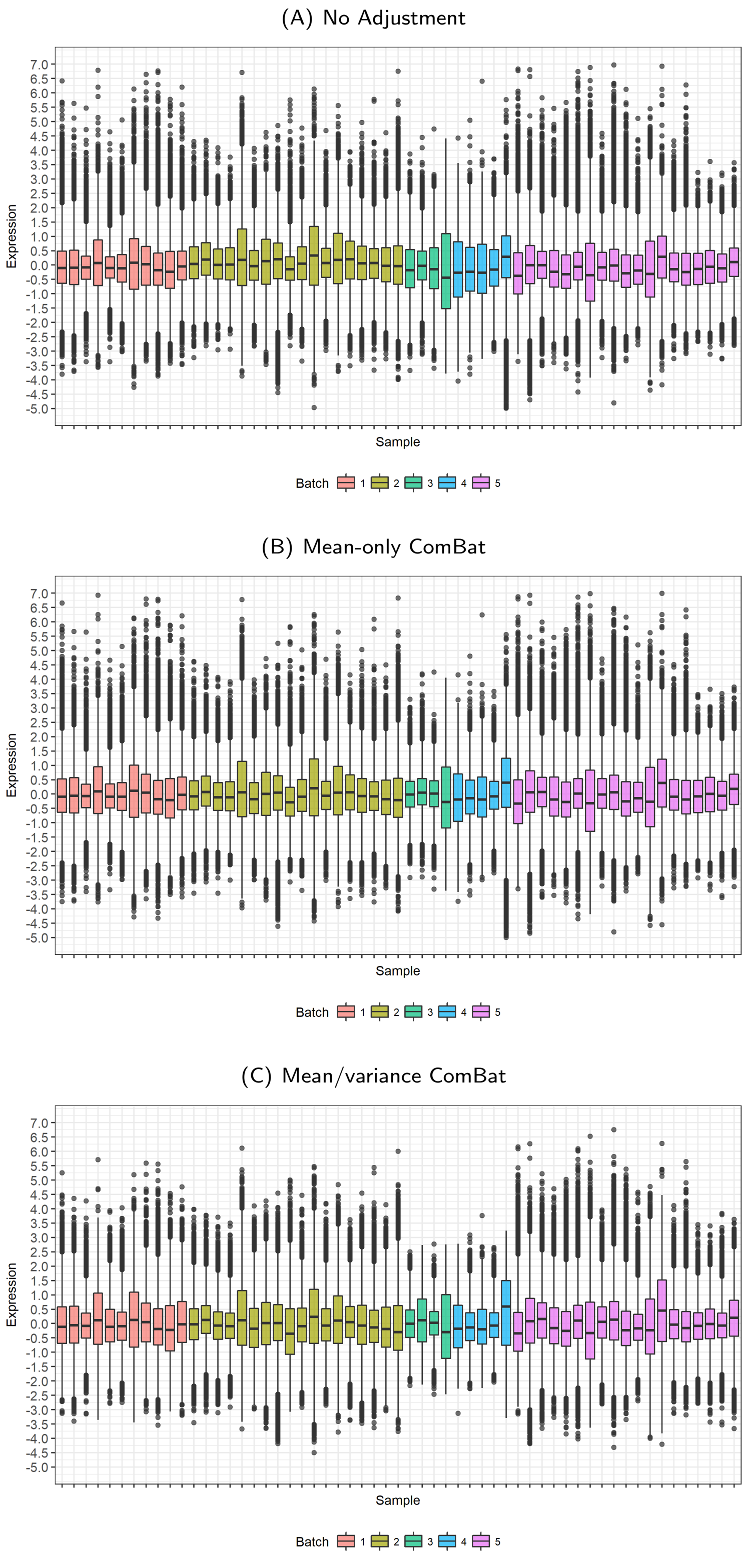

Figure S1: Standardized gene expression in each sample in the bladder cancer dataset. In unadjusted data (A), batch 3 and batch 4 have smaller sample size. The mean-only ComBat (B) does not affect the variance, and results in similar variances in gene expression as in the unadjusted data. (C) The mean/variance ComBat arbitrarily makes gene expression in these two batches less variable, suggesting that it overcorrects the data.

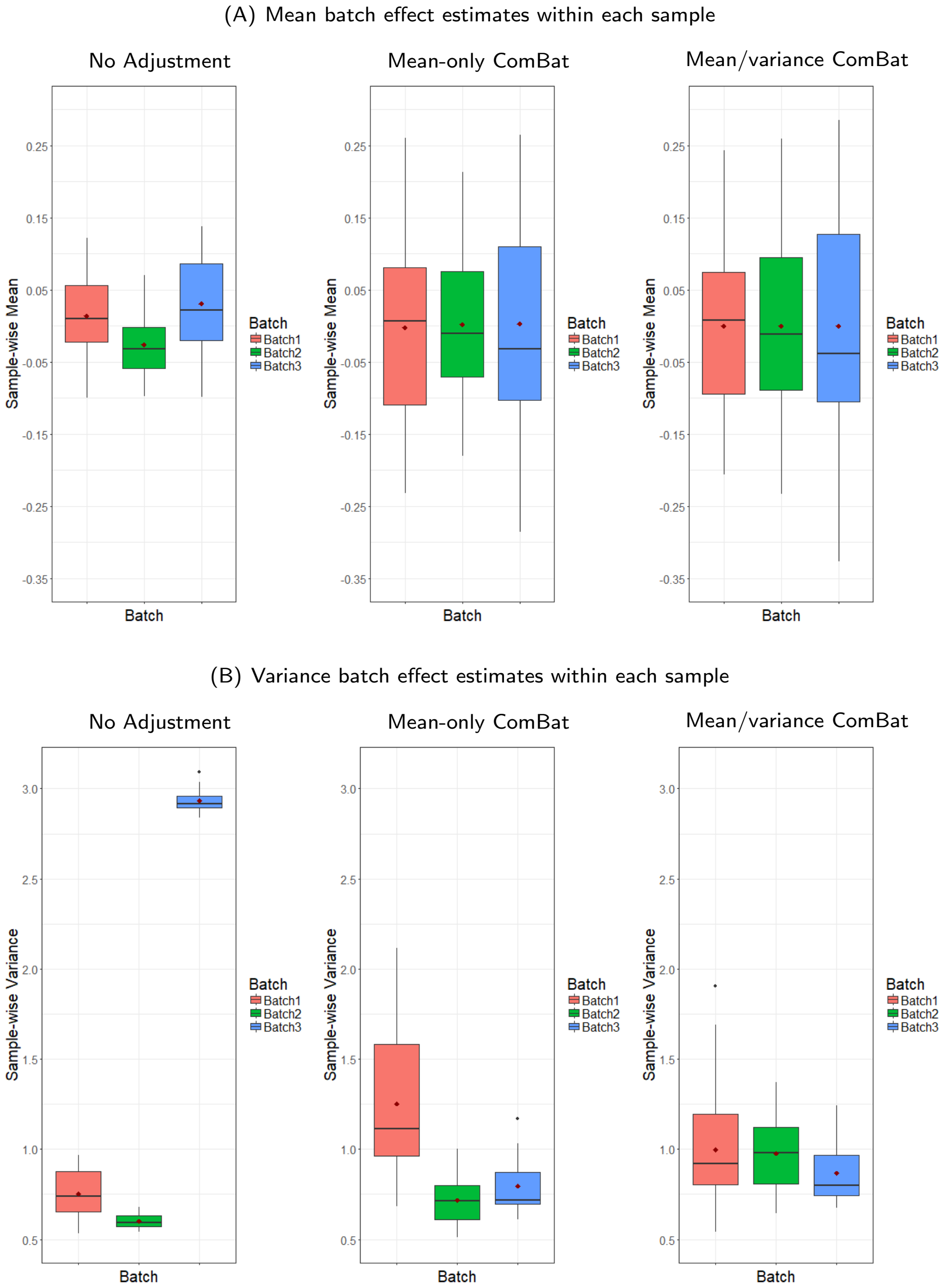

Figure S2: Distribution of sample-wise mean and variance estimates from each batch in the oncogenic signature dataset. Boxplots of sample-wise estimates within each batch, for (A) mean, and (B) variance. Mean-only ComBat is not sufficient to remove the differences in sample-wise variance estimates across batches. Adjusting both the mean and the variance is necessary in this dataset.

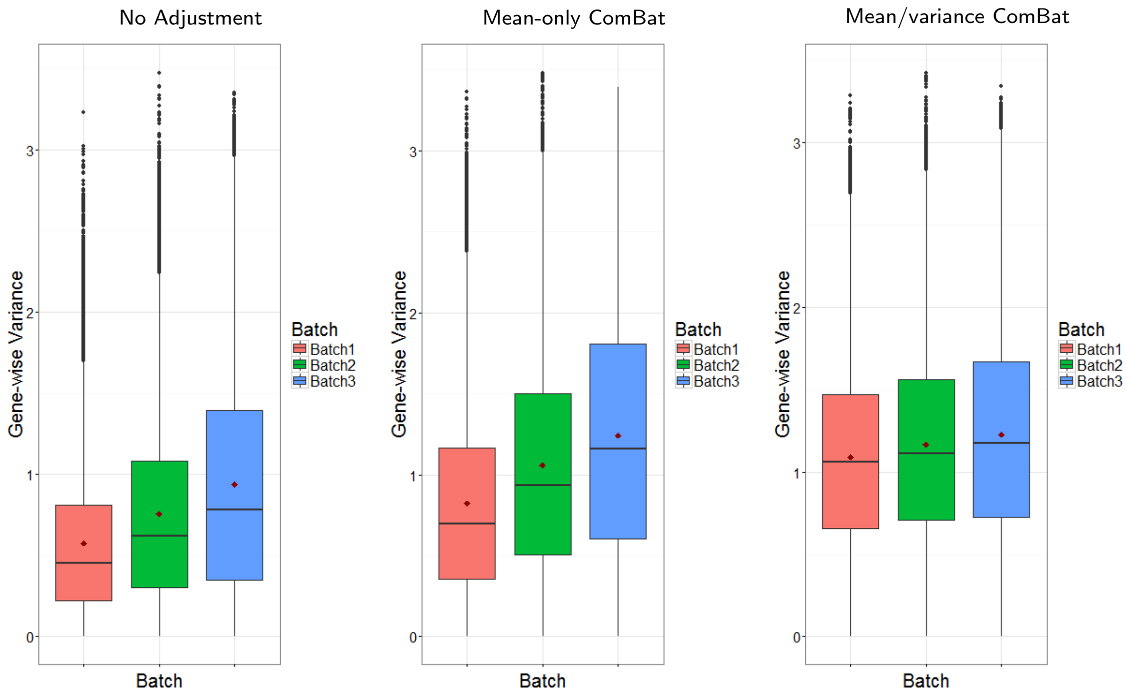

Figure S3: Distribution of gene-wise variance estimates from each batch in the nitric oxide dataset. Only the full mean/variance ComBat can remove the batch effect in variance.

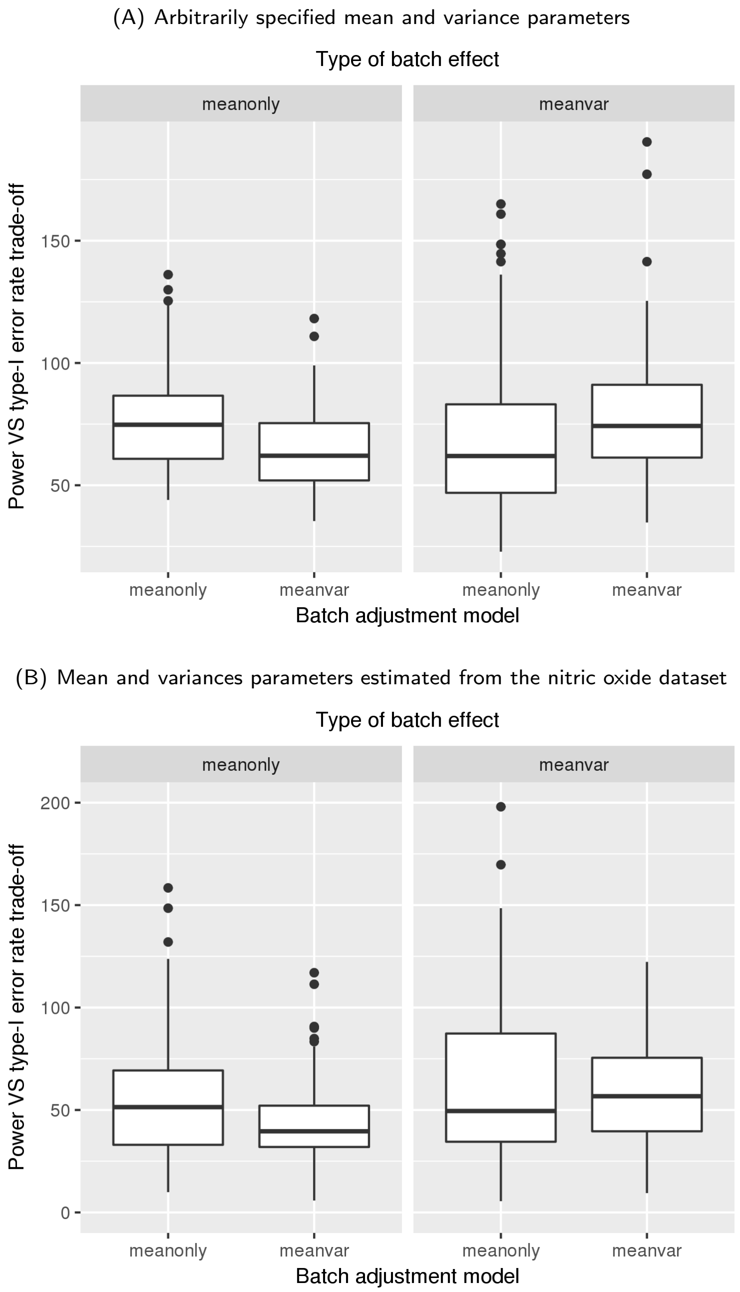

Figure S4: Sensitivity analysis of model selection for batch effect adjustment. We simulated datasets with different types of batch effects ("meanonly": only differences in the batch mean, "meanvar": both mean and variance batch effect). Datasets are simulated from Normal distributions, with parameters that are (A) arbitrarily selected, and (B) estimated from real data. We applied both the mean-only and the mean-variance ComBat to each dataset, and calculated the performance of batch adjustment models using the trade-off score defined by equation 1. Using the model corresponding to the type of batch effect in the data results in better trade-off scores.

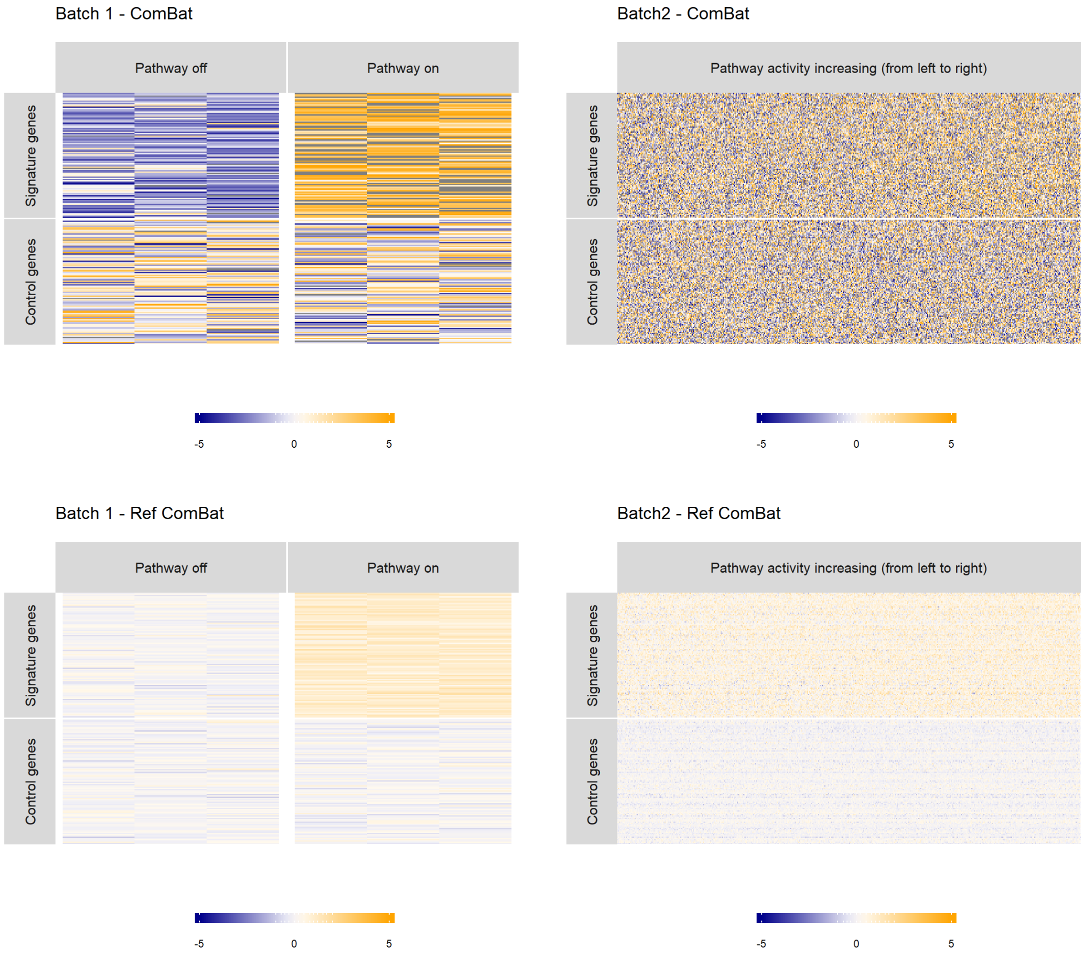

Figure S5: Simulated pathway datasets after batch correction using original and reference-batch ComBat without known activation levels. This figure shows a more realistic setting where the activation levels of pathway are assumed unknown (in both versions of ComBat). Compared with the current ComBat approach, the reference ComBat yields data with better biological signal, even when the pathway activation levels are assumed unknown.

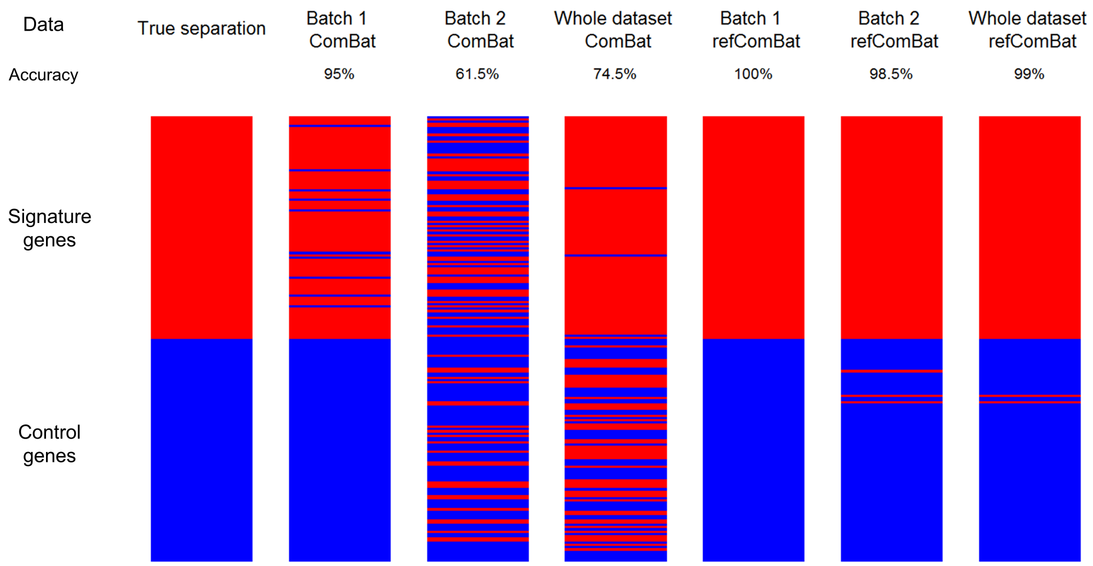

Figure S6: Cluster assignment of the 200 genes using k-means algorithm, assuming unknown activation levels in batch adjustment. Reference-batch ComBat gives cluster assignment that is more consistent with the true separation than original ComBat, in Batch 1 only, Batch 2 only and the combined dataset of Batch 1 and 2. This is consistent with when the two versions of ComBat are used with activation level included as covariates.
